# Eye Injuries from Pencil Lead: Three Cases

**DOI:** 10.4274/tjo.32448

**Published:** 2017-01-17

**Authors:** Ceyhun Arıcı, Osman Şevki Arslan, Burcu Görgülü, Rengin Yıldırım, Umut Onur

**Affiliations:** 1 İstanbul University Cerrahpaşa Faculty of Medicine, Department of Ophthalmology, İstanbul, Turkey; 2 Bakırköy Dr. Sadi Konuk Training and Research Hospital, Ophthalmology Clinic, İstanbul, Turkey

**Keywords:** Intraocular inflammation, pencil lead, intraocular foreign body

## Abstract

Corneal stromal and/or penetrating ocular injuries from pencils and pencil lead are more common in childhood and may lead to intraocular infection or severe intraocular sterile inflammatory reaction. Herein we report 3 children with ocular trauma due to pencil lead injuries. The first case had corneal stromal injury caused by a pencil. In the second case, a pencil perforated the cornea and contacted the iris. In the third case, pencil lead perforated both the cornea and iris and reached the vitreous through the lens zonules. Intracameral triamcinolone (2 mg/0.05 mL) was injected after the pencil lead was removed from the eyeball. Topical anti-inflammatory and cycloplegic drops were prescribed. In conclusion, corneal and especially penetrating ocular injuries from pencil lead may have a good prognosis with the use of appropriate anti-inflammatory and prophylactic antibiotic treatment and follow-up.

## INTRODUCTION

Organic intraocular foreign bodies generally cause serious inflammatory reaction and infection. The inflammatory reaction induced by inorganic foreign bodies is related to the composition of the object.^1^ There are few cases in the literature of intracorneal carbon particles^2,3^ and intraocular penetrating injuries^4,5,6,7,8^ due to pencil lead. Although it has been reported that the carbon particles from pencil lead may remain dormant in the eye without inducing inflammation for long periods of time,^2,3^ they have also been reported to cause severe endophthalmitis5 or endothelial dysfunction and corneal edema.^6^ In this report we share three cases of pencil lead injury, one with corneal stromal injury and two with intraocular penetrating injuries.

## CASE REPORTS

### Case 1

**Figure 1:**
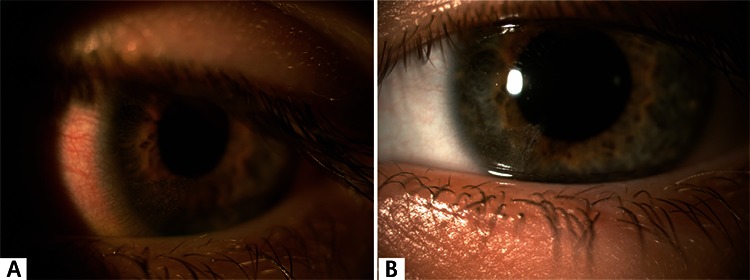
A) Intrastromal carbon particles observed at presentation; B) at four months, the carbon particles are still present but are inert.

An 8-year-old boy was admitted to our urgent ophthalmology clinic the same day of a pencil injury to his left eye. His visual acuity was 20/20 bilaterally. Slit-lamp examination of the left eye revealed intact corneal epithelium and intrastromal silver-gray carbon particles in the inferonasal quadrant of the cornea. The conjunctiva was mildly hyperemic. There was no sign of anterior chamber reaction. Anterior segment examination of the right eye was normal. Intraocular pressure measured by applanation tonometry was 14 mmHg in the right and 17 mmHg in the left eye. Fundus examination was normal in both eyes. The patient was administered moxifloxacin ophthalmic drops (Vigamox 0.5%; Alcon Laboratories Inc., Fort Worth, TX, USA) 4 times a day for 3 weeks and preservative-free artificial tears (Tears Naturale Free; Alcon Laboratories Inc., Fort Worth, TX, USA) 4 times a day for 4 weeks. No signs of inflammatory reaction or injection were observed in follow-up examinations at 1 week and 1, 4, and 6 months (Figures 1A and 1B).

### Case 2

**Figure 2:**
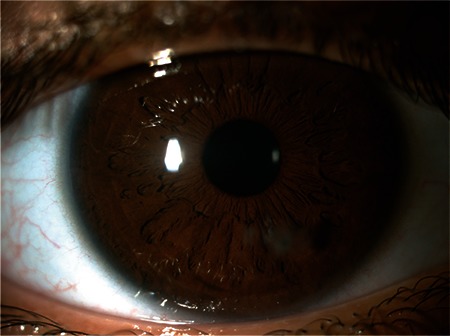
No signs of ocular toxicity are observed in examination at postoperative 7 months

A 13-year-old patient presented after being poked in the right eye by a pencil. The patient’s visual acuity was counting fingers (CF) from 0.5 m in the right eye and 20/20 in the left eye. On slit-lamp examination, a fragment of pencil lead was found lodged in the inferonasal (at 5 o’clock) paracentral cornea of the right eye. Seidel test was negative. Anterior chamber examination revealed +2 cells in the right eye and was normal in the left eye. No signs of pathology were observed in fundus examination of either eye. The patient was admitted for surgical removal of the foreign body. The pencil lead fragment lodged in the cornea was removed using forceps and viscoelastic to support the anterior chamber. The corneal laceration was closed with 4 sutures using 10/0 nylon suture. After the procedure, 2 mg/0.05 mL triamcinolone and 0.25 mg/0.05 mL moxifloxacin were injected into the anterior chamber. On postoperative day 1, treatment was initiated with moxifloxacin drops (6 times a day for 1 week, then reduced to 4 times a day for 2 weeks). Dexamethasone ophthalmic drops (Maxidex, 0.1%; Alcon Laboratories Inc., Fort Worth, TX, USA) were started at 8 times a day for 5 days, then reduced to 6/day for 1 week, then reduced to 4/day for 1 week and tapered by 1 application/day each week until discontinuation. On postoperative day 3, visual acuity on Snellen chart was 20/25 (with or without correction). No inflammatory reaction was observed on anterior segment examination. The corneal sutures were removed at 3-month follow-up. At 7 months, the patient’s visual acuity was 20/20 bilaterally (Figure 2). Corneal scar and a few isolated carbon particles were observed in the inferonasal paracentral cornea of the right eye. There were no further changes noted at 1-year follow-up.

### Case 3

A 15-year-old male patient whose right eye was stabbed with pencil lead was referred from outside the city to our clinic on the second day after the trauma. His visual acuity was CF from 0.5 m on the right and 20/20 on the left. Slit-lamp examination revealed pencil lead in the right temporal cornea (between 8-9 o’clock) that had perforated the cornea and iris near the limbus. Cyclitic membrane was noted around the pupillary region and hypopyon was present (Figure 3A). The left eye appeared normal. Fundus examination could not be performed in the right eye, but was normal in the left eye. The vitreous and retina appeared normal on B-mode ultrasonography of the right eye. The patient was admitted for surgery to remove the foreign body. Under viscoelastic support of the anterior chamber, microforceps were used to remove a foreign body approximately 5.5 mm long and 1 mm in diameter (Figure 3B). After the procedure, 2 mg/0.05 mL triamcinolone and 0.25 mg/0.05 mL moxifloxacin were injected into the anterior chamber. On postoperative day 1, treatment was initiated with moxifloxacin drops (6 times a day for 1 week, then reduced to 4 times a day for 2 weeks); dexamethasone ophthalmic drops were initiated at 8 times a day for 5 days, then reduced to 6/day for 1 week, then 4/day, and finally tapered by 1 application/day each week until discontinuation. The patient’s visual acuity was 20/20 on postoperative day 5. His visual acuity was still 20/20 at 1-year follow-up. On slit-lamp examination, corneal scar tissue and intrastromal carbon particles were noted in the right temporal area. No anterior chamber reaction was observed (Figure 3C).

## DISCUSSION

Pencil lead is made of a mixture of carbon, clay and animal fat and is surrounded by a wooden sheath. The main component, carbon, is known to usually remain inert in the eye. However, potential toxicity due to the other components is controversial.^5,9^ The first reported case of intracorneal carbon particles was presented by Jeng et al.^2^ The patient presented due to a chemical injury to the right eye and silver-gray crystalline opacities were observed in the corneal stroma. It was learned that the patient had sustained a pencil injury to the left eye 8 years earlier. However, the patient’s medical records indicated that the injury had in fact been to the right eye. Slit-lamp examination revealed intact corneal epithelium and silver carbon particles in the inferonasal stroma. This demonstrated that carbon particles in the corneal stroma were well tolerated in the long term. Philip et al.^3^ reported a case in which intracorneal carbon particles were observed during routine eye examination in a patient who had sustained a pencil injury to the same eye 3 years earlier. Slit-lamp examination of the right eye revealed anterior stromal scar, though no signs of previous or current inflammation were detected in the intraocular structures.

There are also reports in the literature of pencil lead causing severe inflammatory reaction and endophthalmitis. One reported case underwent corneal suturation and lens extraction following a pencil injury to the right eye. Pencil lead fragments were noted in the vitreous and on the second postoperative day the patient developed endophthalmitis. Although bacterial endophthalmitis was suspected based on clinical findings, a vitreal sample taken during pars plana vitrectomy was culture negative. It was proposed that the wood and aluminum found in pencils may have caused a severe inflammatory reaction.^5^ In another case with a history of pencil injury, a suspected conjunctival melanoma was excised and the histopathologic report indicated granulomatous reaction due to carbon particles.^10^

Another patient who had sustained a pencil injury to the left eye 4 months earlier presented to an ophthalmologist with a complaint of pain in the left eye for 2 days. Examination revealed a full-thickness corneal scar, a small area of iris atrophy, and a black foreign body resembling pencil lead in the anterior chamber. No inflammatory reaction was observed in the anterior chamber and surgery was performed to remove the foreign body.^9^ No anterior chamber inflammation occurred during the 1-month follow-up period (with tapering topical steroid and cycloplegic agent as medical therapy). A case reported by Gül et al.^8^ presented with severely reduced vision (CF at 2 m) following ocular trauma by pencil. On slit-lamp examination, corneal perforation and fragments of pencil lead were observed at the wound site. The +4 anterior chamber reaction observed preoperatively continued after corneal suturation and foreign body extraction. Examination on the same day revealed linear carbon accumulation on the endothelial surface, and a pencil lead fragment was visible on the lens after pupil dilation. With hourly steroid therapy, the anterior chamber reaction resolved and the endothelial accumulation and material on the lens disappeared. Han et al.^6^ reported a patient with a pencil injury 12 years earlier who presented with stromal keratitis. Antiviral and anti-inflammatory therapy was initiated for a preliminary diagnosis of herpetic stromal keratitis. The patient showed improvement of clinical findings, but at 3-month follow-up, a previously unnoticed foreign body was observed at the anterior chamber angle. The authors believed that the previously inert pencil lead fragment came into contact with the endothelium when it moved, thus triggering an inflammatory reaction. Pencil lead perforated the cornea in our second case and in the third case it perforated both the cornea and iris, reaching the vitreous through the zonules. To prevent a possible inflammatory reaction induced by pencil lead in these patients, triamcinolone was injected into the anterior chamber at the end of surgery, which may be considered possibly beneficial in such cases.

## CONCLUSION

It can be concluded that carbon particles in the cornea are well tolerated in the long term, and that a good prognosis can be achieved in cases of intraocular pencil lead injury with anti-inflammatory therapy, prophylactic antibiotic therapy, and monitoring.

### Ethics

Informed Consent: It was taken.

Peer-review: Externally peer-reviewed.

## Figures and Tables

**Figure 3:**
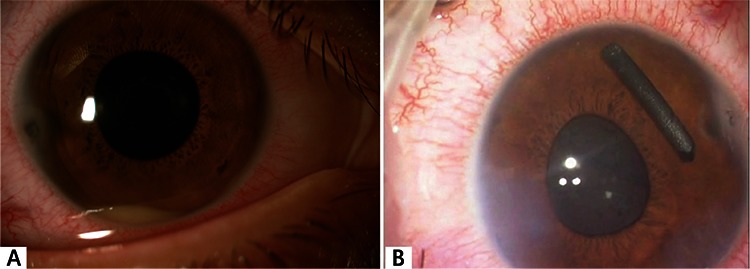
A) At presentation, a foreign body is observed penetrating the cornea in the temporal quadrant. Cyclitic membrane and hypopyon are apparent in the pupillary region; B) a pencil lead fragment that paralimbally perforated the cornea and iris and reached the vitreous through the lens zonules is brought into the anterior chamber during the extraction procedure

**Figure 3C:**
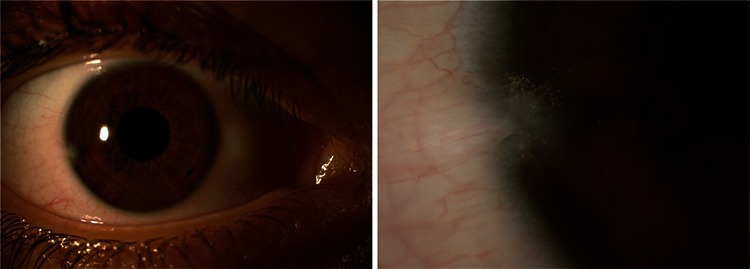
At 1 year, the intrastromal carbon particles are found to be inert and there are no signs of ocular toxicity
